# Helical TomoTherapy versus sterotactic Gamma Knife radiosurgery in the treatment of single and multiple brain tumors: a dosimetric comparison

**DOI:** 10.1120/jacmp.v11i4.3245

**Published:** 2010-07-02

**Authors:** Tushar Kumar, Joseph Rakowski, Bo Zhao, Mazin Alkhafaji, Jacob Burmeister, Tammy Austin, Maria Vlachaki

**Affiliations:** ^1^ Wayne State University School of Medicine Detroit, and Karmanos Cancer Center Detroit MI USA

**Keywords:** Gamma Knife, TomoTherapy, brain tumors

## Abstract

The objective was to compare the dosimetry of Helical TomoTherapy (TOMO) and Gamma Knife (GK) treatment plans for tumor and normal brain in the treatment of single and multiple brain tumors. An anthropomorphic Rando Head phantom was used to compare the dosimetry of TOMO and GK. Eight brain tumors of various shapes, sizes and locations were used to generate 10 plans. The radiation dose was 20 Gy prescribed to the 100% isodose line for TOMO plans and to the 50% for the GK plans. Dose Volume Histograms for tumor and brain were compared. Equivalent Uniform Dose (gEUD), Tumor Control Probability (TCP) and Normal Tissue Complication Probability (NTCP) were performed and used for plan comparisons. Average minimum, mean, median and maximum tumor doses were 19.93, 27.83, 27.38, 39.60 Gy for GK and 20.17, 20.60, 20.59, 20.90 Gy for TOMO. Average gEUD values for tumor and normal brain were 25.0 and 7.2 Gy for GK and 20.7 and 8.1 Gy for TOMO. Conformity indices (CI) were similar for both modalities. Gradient indices (GI) were greater for TOMO. A combination plan was also generated using all eight tumors. TOMO was able to target all eight tumors simultaneously resulting in mean tumor and brain doses of 20.5 and 9.35 Gy, respectively. Due to the maximum limit of 50 beams per plan, GK was unable to provide a treatment plan for all eight tumors. GK provides an advantage for all tumor sizes with respect to tumor and normal brain dose. Clinical studies are needed to correlate these dosimetric findings with patient outcomes.

PACS number: 87.55‐x

## I. INTRODUCTION

Brain metastases occur in 20%–40% of adult patients with cancer, and the incidence of this disease has been increasing approaching 200,000 new cases per year. This increase can be attributed to the earlier diagnosis of the disease due to advances and higher utilization of imaging technology. In addition, prolonged patient survival due to the development of more effective systemic therapies has also been associated with the higher incidence of brain metastasis.^(^ ^1^ ^)^

Treatment options for brain metastases include surgical resection, whole brain radiation therapy, and stereotactic radiosurgery. Clinical trials have demonstrated that stereotactic radiosurgery is an effective first‐line treatment for patients with this disease when compared to surgery or whole brain radiation therapy.^(^ ^2^ ^–^ ^4^ ^)^ In addition, stereotactic radiosurgery has been used for the treatment of recurrences after whole brain radiation therapy.^(^ ^1^ ^,^ ^5^ ^)^ The radiobiological advantage of stereotactic radiosurgery relies on its ability to deliver single, very high‐dose fractions in a small volume with submillimeter accuracy. It is hypothesized that the effectiveness of stereotactic radiosurgery is partially related to the phenomenon of tumor repopulation, which is minimized with such high radiation doses. Moreover, stereotactic radiosurgery achieves a sharp dose gradient between tumor and normal brain, therefore minimizing the risk of radiation‐induced brain toxicity.^(^ ^6^ ^–^ ^8^ ^)^

Stereotactic radiosurgery can be delivered using various equipment and techniques including Gamma Knife, linacs, CyberKnife and more recently Helical TomoTherapy.^(^ ^9^ ^,^ ^10^ ^)^ These techniques differ regarding a number of treatment planning and treatment delivery parameters including the source of radiation beams, the method of beam delivery (coplanar versus non‐coplanar beams), the quality assurance processes, and the resultant tumor and normal tissue dosimetry. The longest experience in the field of stereotactic radiosurgery has been with Gamma Knife (GK) that uses a non‐coplanar concentric array of 201 fixed, sharply collimated cobalt‐60 beams of four discrete isocenter transverse diameters: 4, 8, 14 and 18 mm. However, most GK models (B and C series) are designed for brain only stereotactic radiosurgery, while occasionally providing limited access to peripheral brain lesions or those located close to the foramen magnum.^(^ ^11^ ^)^

TomoTherapy (TOMO) is also used for the delivery of stereotactic therapy. It seamlessly combines linear accelerator and megavoltage computed tomography capabilities for verifications of patient and tumor positioning. Using IMRT treatment planning, it generates a helical fan beam around the patient that is further modulated by a binary multileaf collimator, resulting in a highly conformal dose distribution. TOMO is currently used for stereotactic body radiation therapy and fractionated stereotactic therapy for brain tumors. However, its use as a device for stereotactic brain radiosurgery has not yet been established, and data comparing the dosimetry of TOMO and GK is limited.^(^ ^12^ ^)^

At our institution, we have available both Gamma Knife C series (Elekta Inc., Norcross, GA) and Helical TOMO (TomoTheraphy Inc., Madison, WI), and we conducted a dosimetric phantom study to assess whether dosimetric equivalency between these two technologies can be achieved for the treatment of single and multiple brain metastases. In this study, we used eight tumors of various sizes, shapes and locations within the phantom. Dose‐volume statistics were generated and compared for GK and TOMO plans. Due to the presence of dose inhomogeneities in tumor and normal brain tissue, we compared Equivalent Uniform Dose (gEUD), Tumor Control Probability (TCP) and Normal Tissue Complication Probability (NTCP) calculations. Conformity Indices were also evaluated.

## II. MATERIALS AND METHODS

For the purpose of this study, an anthropomorphic Rando Head phantom was used. A 40‐slice CT simulation scanner (Siemens SOMATOM Sensation Open) was used to acquire the phantom CT images. The images were subsequently electronically transferred to both the Gamma Knife treatment planning system, and ECLIPSE treatment planning system (Varian). Eight tumor contours of various sizes, shapes and locations as well as normal brain contours were generated in both planning systems. Regarding tumor contours, there were six oblate spherical and two irregularly shaped lesions, with the largest diameters ranging from 7 mm to 40 mm in size. Three out of eight lesions were centrally located within the phantom. For TOMO treatment planning, the contours were electronically transferred from ECLIPSE to TOMO treatment planning system. A total of 10 plans were generated: five for single peripheral tumors, three for centrally located tumors, and one combining three lesions (two peripheral and one central). An additional plan was generated using all eight lesions with TOMO only. An attempt to generate a similar plan with GK failed because each GK plan is limited to a maximum of fifty isocenters, and many isocenters were needed to achieve high conformality to the oddly shaped lesions. For the purposes of this study, the prescribed dose was 20 Gy for all tumors, prescribed to the 100% isodose line for TOMO and 50% isodose line for GK plans.

Dose Volume Histograms (DVHs) for tumor and normal brain were generated and compared for both GK and TOMO plans. Since significant dose inhomogeneity is observed for tumor with GK and for brain with both technologies, biological models including gEUD, TCP and NTCP were utilized to assess the biological effectiveness of these plans. In calculating gEUD, it is assumed that two different target dose distributions are equivalent if the corresponding number of expected surviving clonogens is equal. The following formula was used to calculate gEUD: ^(^ ^13^ ^)^

(1) $$\text{gEUD}={\left(\frac{1}{N}\sum_{i=1}^{N}d_{i}^{a}\right)}^{1/a}$$

where $d_{i}$ represents the dose to voxel *i*, *N* corresponds to the number of voxels, and *a* equals the number of single hit events in the linear quadratic model of cell killing (‐10 for tumor and 5 for the brain).

In calculating TCP, it is assumed that the local tumor control is achieved when all the clonogenic cells are destroyed by radiation using the linear quadratic expression for cell killing and Poison statistics.^(^ ^14^ ^–^ ^18^ ^)^ It was calculated using the following formula:

(2) $$\text{TCP}=e^{‐N{\left(SF2\right)}^{\frac{D}{Dref}\cdot \frac{{\;}^{\alpha }{/}_{\beta }+{\;}^{D}{/}_{n}}{{\;}^{\alpha }{/}_{\beta }+Dref}}}$$

where *N* is the number of clonogenic cells in the tumor equaling $10^{7}/$ cubic centimeter of tumor volume. The α and β are radiosensitivity parameters related to cell killing from single or multiple hit events respectively and their ratio equals to 10. $SF_{2}$ is the cell surviving fraction after irradiation at reference dose of $2\;\text{Gy}(D_{\text{ref}})$, *D* equals gEUD, and *n* equals the number of treatment fractions.

Finally, NTCP is the probability that a percentage of the patient population will incur a persistent detrimental brain late effect after receiving a particular radiation dose. As in TCP, NTCP is graphically depicted as a sigmoidal curve as a function of radiation dose and is calculated by the Sigmoidal Dose Response (SDR) NTCP model:^(^ ^19^ ^–^ ^21^ ^)^

(3) $$\text{NTCP}=\Phi \left(\frac{EUD‐D_{50}}{mD_{50}}\right)$$

where Φ(x) is the probit function:

(4) $$\Phi (x)=\frac{1}{\sqrt{2\pi }}\int_{‐\infty }^{x}\exp\left(\frac{‐t^{2}}{2}\right)dt=\frac{1}{2}\left[1+erf\left(\frac{x}{\sqrt{2}}\right)\right]$$

and $D_{50}$ corresponds to the dose that will result in necrosis/infarction in 50% of patients.

Gafchromic film dosimetry was performed for two lesions to assess the feasibility of TOMO stereotactic radiosurgery dose delivery.^(^ ^22^ ^)^ The smallest lesion of 7 mm in size and the centrally located irregular lesion were chosen for this study. A film gamma analysis (i.e. composite of dose difference and distance to agreement) was also performed.

## III. RESULTS

Tables 1(a) and 1(b) describe the treatment planning parameters for TomoTherapy and Gamma Knife. Compared to TOMO plans, treatment times were considerably longer for GK (Table 1(c)). GK plans resulted in higher tumor doses and increased tumor dose inhomogeneity. Average minimum, mean, median and maximum tumor doses were 19.93, 27.83, 27.38, 39.60 Gy for GK, and 20.17, 20.60, 20.59, 20.90 Gy for TOMO (Table 2). GK plans resulted in lower doses to 1%, 5%, 15% and 25% normal brain volumes, dose differences ranging from 0.09 to 6.54 Gy, with the exception of the 10 mm lesion at 25%, peripheral irregular lesion at 25% and central irregular lesion at 1% and 25%, for which TOMO delivered a lower normal brain dose (Table 3). Figure 1(a)‐(i) illustrates the dose volume histograms for all eight plans. A combination plan was generated with TOMO using all eight tumors. TOMO was able to target all eight tumors simultaneously resulting in mean tumor and brain doses of 20.5 and 9.35 Gy, respectively. Due to the maximum limit of 50 beams per plan, we were unable to complete a treatment plan that includes all eight tumors with GK.

**Table 1(a):** Treatment planning parameters for TomoTherapy.

*Lesion Size*	*Grid*	*Pitch*	*Std. Dev.*	*Field Width*
7 mm	Fine	0.08	0.06	1.05 cm ‐ Jaws(0.35‐0.35)
10 mm	Fine	0.08	0.11	1.05 cm ‐ Jaws(0.35‐0.35)
20 mm	Fine	0.08	0.15	1.05 cm ‐ Jaws(0.35‐0.35)
20 mm Central	Fine	0.08	0.14	1.05 cm ‐ Jaws(0.35‐0.35)
30 mm	Fine	0.08	0.14	1.05 cm ‐ Jaws(0.35‐0.35)
40 mm	Fine	0.08	0.1	1.05 cm ‐ Jaws(0.35‐0.35)
Irreg 1	Fine	0.08	0.17	1.05 cm ‐ Jaws(0.35‐0.35)
Irreg 2	Fine	0.08	0.14	1.05 cm ‐ Jaws(0.35‐0.35)
3 Lesions (20, 20c, 30)	Fine	0.08	0.22	1.05 cm ‐ Jaws(0.35‐0.35)
8 Lesions	Fine	0.08	0.25	1.05 cm ‐ Jaws(0.35‐0.35)

**Table 1(b):** Tomo Modulation Factor, Tomo Weighting, GK # of isocenters and cone size

*Tomo Weighting*						
*Lesion Size*		*Tomo Modulation Factor*	*Max Dose Penalty*	*Min Dose Penalty*	*GK # of Isocenter*	*GK Cone Size*
7mm	CTV Brain	1.582 ‐	1 ‐	1 ‐	3 ‐	4 mm, 8 mm ‐
10mm	CTV Brain	1.994 ‐	1 ‐	1 ‐	10 ‐	4 mm ‐
20mm	CTV Brain	2.004 ‐	1 ‐	1 ‐	24 ‐	4 mm ‐
20mm Central	CTV Brain	2.002 ‐	35 ‐	1 ‐	18 ‐	4 mm, 8 mm ‐
30mm	CTV Brain	1.999 ‐	1 ‐	1 ‐	28 ‐	4 mm, 8 mm, 18 mm ‐
40mm	CTV Brain	2.001 ‐	1 ‐	1 ‐	40 ‐	4 mm, 8 mm, 14 mm ‐
40 mm Central	CTV Brain	1.997	30 ‐	1 ‐	31	4 mm, 8 mm, 14 mm
Irreg 1	CTV Brain	1.974 ‐	30 ‐	1 ‐	37 ‐	4 mm, 8 mm ‐
Irreg 2	CTV Brain	1.998 ‐	30 ‐	1 ‐	32 ‐	4 mm, 8 mm ‐
3 Lesions (20, 20c, 30)	CTV Brain	1.002 ‐	20 ‐	1 ‐	50 ‐	4 mm, 8 mm ‐
8 Lesions (7, 10, 20, 20c, 30, 40, Irreg 1, Irreg 2)	CTV Brain	1.989 ‐	25 ‐	5 ‐	‐	‐

**Table 1(c):** Treatment delivery times for TomoTherapy and Gamma Knife.

*Lesion Size*	*Tx. Time Tomo (seconds)*	*Tx. Time GK (seconds)* ^a^
7 mm	656.5	3579
10 mm	1303.3	5940
20 mm	1287.5	12037
20 mm Central	1283.7	10333
30 mm	2187.7	15382
40 mm	2189.1	13505
Irreg 1	1364.7	19763
Irreg 2	1087	21965
3 Lesions (20, 20c, 30)	6192.9	29237
8 Lesions	5666.7	‐

a For July 29, 2009 at a dose rate of 1.609 Gy/min for the 18 mm collimator. The full strength date for the sources is 7/18/03 at 3.562 Gy/min.; the half life is 1925.28 days.

**Table 2:** Minimum, mean, median and maximum Dose (Gy).

*Tumor Size (mm)*	*Minimum Dose (Gy)*		*Mean Dose (Gy)*		*Median Dose (Gy)*		*Maximum Dose (Gy)*	
*Peripheral*	*GK*	*TOMO*	*GK*	*TOMO*	*GK*	*TOMO*	*GK*	*TOMO*
7	20.80	20.00	30.33	20.18	29.90	20.18	39.60	20.25
10	21.20	20.20	29.44	20.50	28.64	20.48	39.60	20.70
30	19.60	20.20	28.60	20.63	28.00	20.60	39.60	21.00
40	19.20	20.15	26.34	20.57	25.56	20.57	39.60	20.85
Irregular	20.80	20.20	27.59	20.70	27.05	20.72	39.60	21.00
*Three Lesions*								
20	19.20	20.15	27.68	20.59	27.17	20.58	39.60	20.90
central 20	19.60	20.10	26.92	20.58	26.53	20.60	39.60	20.95
30	19.60	20.05	30.36	20.54	30.66	20.51	39.60	20.90
*Central*								
20	20.80	20.50	27.11	20.90	26.88	20.90	39.60	21.25
40	18.80	20.15	25.67	20.61	25.30	20.60	39.60	20.85
Irregular	19.60	20.20	26.11	20.79	25.50	20.72	39.60	21.20
*Mean*	19.93	20.17	27.83	20.60	27.38	20.59	39.60	20.90

**Table 3:** Brain dose volume differences in Gy. A positive number indicates that TomoTherapy delivers a higher dose than Gamma Knife.

*Brain%Vol.*		*Peripheral Tumor (mm)*				*Central Tumor (mm)*			*Peripheral and Central Tumors (mm)*
	*7*	*10*	*30*	*40*	*Irregular*	*20*	*40*	*Irregular*	*20+30+ Central 20*
1%	3.85	4.43	5.71	5.28	4.83	6.54	3.14	‐1.65	3.26
5%	1.75	2.07	3.13	4.90	2.81	3.08	2.89	1.44	3.56
15%	0.80	0.8	1.46	2.58	1.00	1.45	1.19	0.87	2.24
25%	0.09	‐0.22	0.46	0.45	‐0.31	0.62	0.55	‐0.21	1.42

**Figure 1(a):**
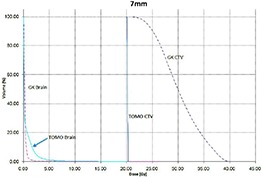
DVH of 7mm lesion.

**Figure 1(b):**
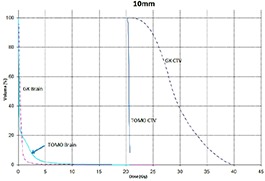
DVH of 10 mm lesion.

**Figure 1(c):**
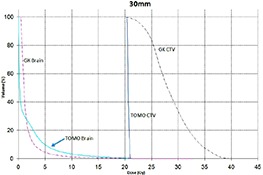
DVH of 30 mm lesion.

**Figure 1(d):**
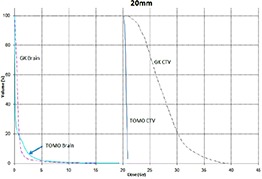
DVH of 20 mm lesion.

**Figure 1(e):**
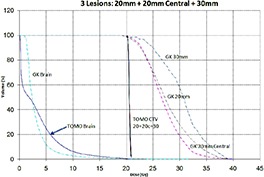
DVH of three lesions (20 mm, 20 mm central, 30 mm).

**Figure 1(f):**
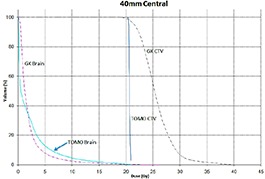
DVH of 40 mm central lesion.

**Figure 1(g):**
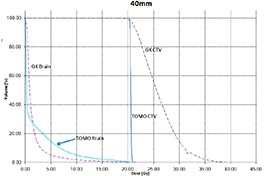
DVH of 40 mm peripheral lesion.

**Figure 1(h):**
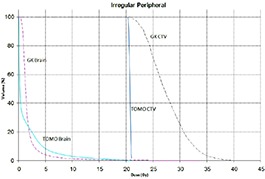
DVH of irregular peripheral lesion.

**Figure 1(i):**
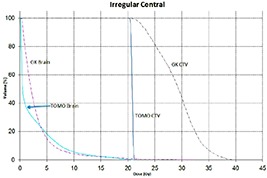
DVH of irregular central lesion.

The gEUD calculations for tumor and normal brain also demonstrated that GK delivers higher doses to the lesions compared to TOMO, with overall mean of 25.0 versus 20.7 Gy, respectively. Regarding normal brain gEUD, TOMO uniformly delivered higher doses compared to GK, with overall mean of 8.1 versus 7.2 Gy, respectively (Table 4).

**Table 4:** gEUD

*Tumor Size (mm)*	*GK*		*TOMO*	
*Peripheral*	*Tumor*	*Brain*	*Tumor*	*Brain*
7	27.3	3.9	20.7	4.7
10	26.8	4.7	20.6	5.5
30	25.9	7.1	20.6	9.7
0	23.7	7.3	20.6	8.9
Irregular	25.3	7.8	20.7	8.0
*Three Lesions*				
20	24.6	8.5	20.6	9.1
Central 20	24.6	8.5	20.6	9.1
30	26.6	8.5	20.5	9.1
*Central*				
20	22.4	6.2	20.9	6.9
40	24.4	8.0	20.6	8.8
Irregular	23.9	8.8	20.8	9.1
*Mean*	25.0	7.2	20.7	8.1

Due to the higher TCP associated with the GK plans, we determined TOMO gEUD values that would produce GK equivalent TCP values. The TOMO plans, however, were not reoptimized to a higher prescription dose. A TCP equalization factor, called the “TOMO Factor”, was calculated as the ratio (TOMO gEUD to achieve GK equivalent TCP)/(planned TOMO gEUD). Applying this ratio to the TOMO tumor and normal brain gEUD values produced an increase in the average tumor and normal brain gEUD to 25.0 and 9.7 Gy, respectively. The Tomo Factors ranged from 1.07 to 1.30 (Table 5). Table 6 shows the tumor and normal brain gEUD values for the corrected TOMO dose distributions. These were derived by multiplying each individual tumor and normal brain gEUD by the Tomo Factor. In the equalized TOMO plans, the mean tumor and normal brain gEUD values increased by 4.3 Gy and 1.6 Gy, respectively. Similarly, the TCP values were also corrected using the TOMO factor, as seen in Table 7.

**Table 5:** Corrected gEUD for TomoTherapy.

*Tumor Sze (mm)*		*Tumor Dose (Gy)*	
*Peripheral*	*Original TOMO gEUD*	*TOMO gEUD to Achieve GK Equivalent TCP*	^a^ *TOMO Factor*
7	20.7	26.8	1.29
10	20.6	26.8	1.30
30	20.6	25.95	1.26
40	20.6	23.73	1.15
Irregular 2	20.7	25.39	1.23
*Three Lesions*			
20	20.6	24.6	1.19
Central 20	20.6	24.65	1.18
30	20.5	26.65	1.30
*Central Lesions*			
20	20.9	22.46	1.07
40	20.6	23.95	1.16
Irregular 1	20.8	23.98	1.15

a TOMO factor=(TOMOgEUDtoachieveGKequivalentTCP)/(PlannedTOMOgEUD).

**Table 6:** GK gEUD vs. corrected gEUD for TOMO and corrected TCP for TOMO.

*Tumor Location/Size (mm)*	*GK (Gy)*		*TOMO (Gy)*		*TCP Equivalent TOMO (Gy)*	
*Peripheral*	*Tumor*	*Brain*	*Tumor*	*Brain*	*Tumor*	*Brain*
7	27.3	3.9	20.7	4.7	26.80	6.1
10	26.8	4.7	20.6	5.5	26.80	7.2
30	25.9	7.1	20.6	9.7	25.95	12.2
40	23.7	7.3	20.6	8.9	23.73	10.2
Irregular	25.3	7.8	20.7	8.0	25.39	9.8
*Three Lesions*						
20	24.6	8.5	20.6	9.1	24.60	10.8
Central 20	24.6	8.5	20.6	9.1	24.65	10.7
30	26.6	8.5	20.5	9.1	26.65	11.8
*Central*						
20	22.4	6.2	20.9	6.9	22.46	7.4
40	24.4	8.0	20.6	8.8	23.95	10.2
rregular	23.9	8.8	20.8	9.1	23.98	10.5
*Mean*	25.0	7.2	20.7	8.1	25.00	9.7

**Table 7:** TCP values for GK, TOMO and corrected TOMO.

*Tumor Location/Size (mm)*	*TCP*		
*Peripheral*	*GK*	*Original TOMO*	*TCP Equivalent TOMO*
7	1.0000	0.9880	1.0000
10	1.0000	0.9643	1.0000
30	0.9999	0.4221	0.9999
40	0.9896	0.2412	0.9896
Irregular	0.9999	0.7514	0.9999
*Three Lesions*			
20	0.9997	0.8330	0.9997
Central 20	0.9997	0.8048	0.9997
30	1.0000	0.3681	1.0000
*Central*			
20	0.9875	0.8703	0.9875
40	0.9930	0.2541	0.9930
Irregular	0.9944	0.4256	0.9944

GK achieved TCP values ranging from 98.75% to 100%. TCP for the noncorrected TOMO plans ranged from 24.1% to 98.8%, while the corrected ‘TCP Equivalent TOMO’ values ranged from 98.75% to 100% (Table 7). NTCP was 0% (on the order of 10‐10 to 10‐8) for all plans.

Conformity indices were calculated according to Paddick's formula:

(5) $$\text{CI}={\left({\text{TV}}_{\text{PIV}}\right)}^{2}/(\text{TV}\;\;x\;\;\text{PIV})$$

where ${\text{TV}}_{\text{PIV}}$ is the volume of tumor encompassed by the Prescription Isodose Volume (PIV), and TV is the tumor volume (Table 8).^(^ ^23^ ^)^ Values can range from 0 for a total miss, to 1 for perfect conformity. Conformity indices ranged from 0.38 to 0.80 for GK, and from 0.46 to 0.73 for TOMO.

**Table 8:** Conformity and gradient indices of GK and TOMO.

*Tumor Location/Size (mm)*				
*Peripheral*	*CI GK*	*CI Original TOMO*	*GI GK*	*GI Original TOMO*
7	0.52	0.52	2.86	17.81
10	0.44	0.46	3.03	10.53
20	0.52	0.68	2.85	4.49
30	0.73	0.67	2.96	4.17
40	0.80	0.69	2.58	4.05
Irregular 2	0.38	0.58	3.23	8.98
*Three Lesions*				
20	0.61	0.51	3.09	7.45
Central 20	0.53	0.56	3.20	5.93
30	0.68	0.73	2.91	5.35
*Central*				
20	0.68	0.54	2.87	6.00
40	0.71	0.67	2.68	3.78
Irregular 1	0.51	0.62	3.19	5.14

Gradient indices (GI) were calculated in Tomo as the ratio of the volume of half the prescription isodose to the volume of the prescription isodose, and in GK as the ratio of the 25% isodose volume to that of the 50% isodose volume (Table 8).^(^ ^24^ ^)^ Gradient indices ranged from 2.58 to 3.23 with GK and 3.78 to 17.81 with Tomo (Table 8).

Comparison of the dose profiles and isodose distributions of TOMO treatment planning and Gafchromic film dosimetry for two lesions verified that TOMO accurately delivers the planned stereotactic radiosurgery doses. Figures 2a–(b) demonstrate dose profiles and isodose distributions of TOMO plan depicted with thick lines superimposed on to the Gafchromic film doses, which are depicted with fine lines. Relative comparison shows that the 7 mm gamma distribution was excellent, with less than 0.1% of the pixels with gamma greater than 1 Fig. 2(a). Analysis of the central irregular lesion was clinically acceptable showing the number of pixels with a gamma value greater than 1 to be less than 10% Fig. 2(b).

**Figure 2(a):**
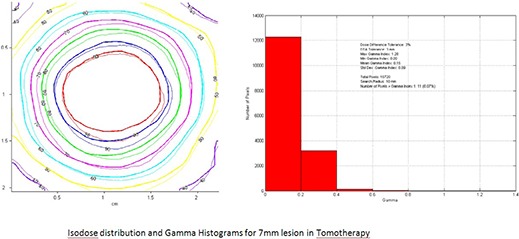
Isodose distribution and gamma histogram of 7 mm lesion in TomoTherapy.

**Figure 2(b):**
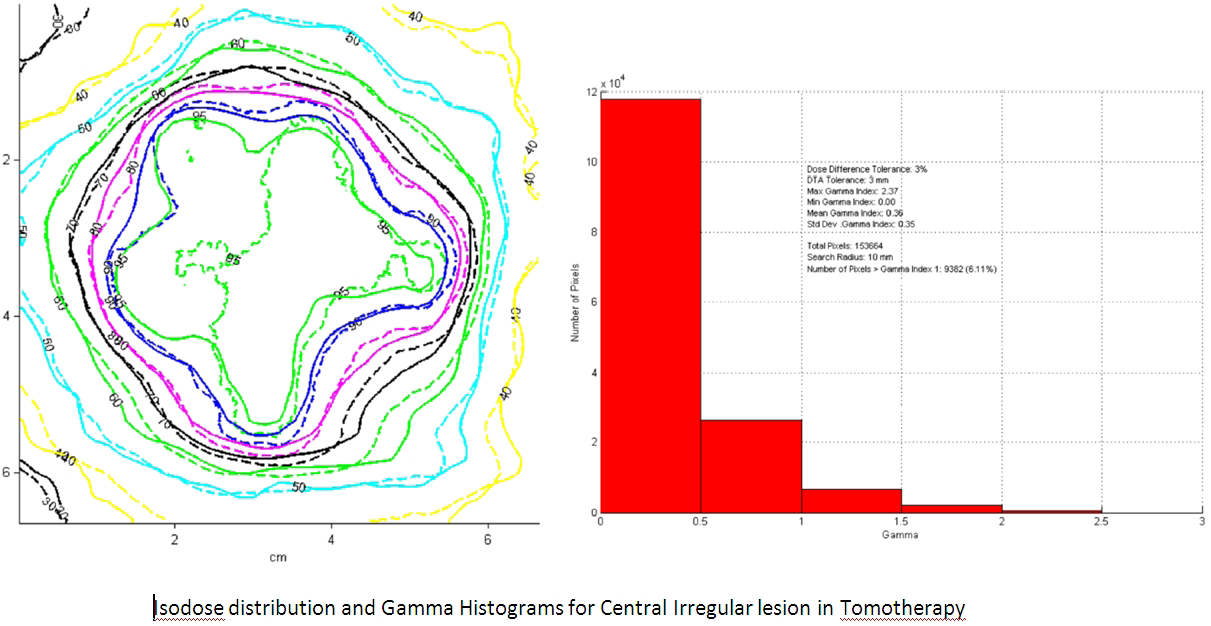
Isodose distribution and gamma histogram of central irregular lesion in TomoTherapy.

## IV. DISCUSSION

In our study, a radiation dose of 20 Gy was prescribed to the periphery of the tumor. Although current practices with linac‐based stereotactic radiosurgery include a 1–2 mm tumor margin to account for setup error, no margin was used for the TOMO plans. This approach was chosen to minimize dosimetric differences arising from having consistently larger targets in the TOMO group of plans. GK resulted in higher tumor median, mean, and maximum doses compared to TOMO by 7.23, 6.79 and 18.70 Gy, respectively. TOMO resulted in higher whole brain gEUDs for all lesions (Figs. 2–4). The GK plans also achieved higher GEUD and TCP values. This is attributed to prescribing to the 50% isodose line with GK versus the 100% with TOMO. Using the “TOMO Factor” to normalize the TOMO plans to achieve TCP values equivalent to those of the GK plans resulted in higher normal brain doses.

The only dosimetric study comparing the dosimetry of GK to TOMO is limited to five patients with single brain metastasis.^(^ ^12^ ^)^ The authors attempted to generate a TOMO plan with tumor dose inhomogeneity comparable to that of a GK plan by incorporating a “simultaneous integrated boost”. Although both techniques had high conformity indices, TOMO resulted in smaller hot spots within the tumor and larger low dose radiation volumes within the normal brain. As a result, the authors concluded that dosimetric equivalency between GK and TOMO could not be attained in this study. The superior tumor and normal tissue dosimetry observed with GK may have potential radiobiologic implications for stereotactic brain radiosurgery.^(^ ^17^ ^)^

GK and linac‐based stereotactic radiotherapy techniques have been used over the past 20 years to treat brain tumors. Significant clinical and dosimetric data comparing these two methods of stereotactic radiotherapy delivery have been published based on the treatment of acoustic neuromas^(^ ^25^ ^–^ ^27^ ^)^ and skull based tumors.^(^ ^28^ ^)^ Early publications demonstrated that GK achieves superior conformality and dose fall‐off at the edge of the target volume as compared to linac‐generated fixed arc, non‐coplanar beams.^(^ ^11^ ^)^ With the introduction of micro‐multileaf collimators (MLC) with central leaves as small as 3 mm in size, the dosimetric superiority of GK has been challenged. In addition, patient immobilization techniques using relocatable frames in linac‐based radiosurgery as opposed to the fixed‐invasive ones used in GK are actively being studied in relation to the accuracy of patient positioning, the planning target volume definition, and the radiation dosing to the target and surrounding normal tissues. Finally, the potential radiobiological impact of the profound differences in target dose inhomogeneity between the two techniques is yet to be uncovered.

Perks et al.^(^ ^25^ ^)^ compared the dosimetric differences between GK and two linac‐based stereotactic radiosurgery techniques utilizing the BrainLAB system (BrainLAB AG, Munich, Germany). The first linac‐based technique employed fixed non‐coplanar beams with static MLC leaves, while the second used a series of arcs where the MLC leaves dynamically moved to adjust to the altering shape of the target projection. This study revealed that GK resulted in superior dose conformality compared to the dynamic arc and fixed beam techniques (conformality index 1.38, 1.65 and 1.78, respectively). GK also delivered lower maximum brainstem doses with the exception of two patients who had the largest tumor volumes (4.15 and 10.61 cc). The authors concluded that the emergence of improving linac‐based stereotactic techniques will compete with GK because of their dosimetric superiority in larger tumors, and their ability to target both intracranial and extracranial lesions – delivering the treatment both in single or multiple fractions.

Dosimetric comparison of linac‐based stereotactic radiotherapy to TOMO was conducted by Soisson et al.^(^ ^28^ ^)^ in ten patients with skull‐base tumors. In this study, fractionated stereotactic radiation therapy was used to a total target dose of 50.4 Gy in 28 fractions. Compared to TOMO, non‐coplanar beam arrangement resulted in improved prescription isodose to target volume ratio (1.44 versus 2.22) and limited radiation dose spillage to uninvolved brain. The authors concluded that non‐coplanar beams offer a dosimetric advantage for the treatment of skull‐base tumors.

Unlike GK, TOMO was able to deliver the desired radiation dose of 20 Gy simultaneously to eight lesions in our study. However, we were unable to generate such plan with GK. GK may only use a maximum of 10 matrices and/or 50 radiation beams for a single treatment plan. Also, depending on the location of the lesions, some GK models may require multiple frame placements in successive procedures to successfully and conformally target all tumors. The mean dose to the whole brain in the eight‐lesion TOMO plan was quite high, at 9.35 Gy. GK has been used extensively to treat multiple brain lesions in staged procedures but the resultant whole brain dose and potential radiobiological effects from such treatment are unknown.^(^ ^29^ ^–^ ^31^ ^)^ As brain toxicity correlates with fraction size, hypofractionated radiotherapy may be preferable for the simultaneous treatment of multiple brain tumors using linac‐based stereotactic techniques. Moreover, with the advent of more effective chemotherapy and targeted therapies, cancer patients with systemic and central nervous system metastases survive longer, thus being subject to a higher risk of manifesting the clinical signs and symptoms of long‐term brain toxicity.

The low dose radiation spillage to the normal brain seen with TOMO may in fact be deemed dosimetrically beneficial in patients requiring whole brain radiation therapy in addition to radiosurgery. It has been used to integrate a simultaneous boost with whole brain radiation therapy in the management of brain metastases. Bauman et al.^(^ ^32^ ^)^ studied this treatment approach in 14 patients with one to three brain tumors. In this study, the uninvolved brain and the metastatic tumors received simultaneously doses of 30 and 60 Gy, respectively. This treatment provides access to frameless stereotaxis and accurate target localization using megavoltage CT. Moreover, additional radiobiologic advantage may be derived from combining, instead of sequencing, whole brain radiation therapy and stereotactic boost.

The incidence of radiation necrosis in patients with brain metastases treated with stereotactic radiosurgery ranges from 5%–10% within the first two years of treatment,^(^ ^33^ ^–^ ^37^ ^)^ and is histologically characterized by fibrinoid necrosis and hyalinization of blood vessels as well as accumulation of inflammatory cells within and in the rim of the necrotic lesion.^(^ ^38^ ^)^ Many investigators believe that the mechanism of necrosis involves a chromic inflammatory process mediated by cytokine production. It is unclear, though, whether the endothelial cell or the glial cell is the responsible for initializing the inflammatory process associated with radiation injury.^(^ ^39^ ^–^ ^40^ ^)^ Factors related to radiation necrosis include radiation dose and dose per fraction, treatment volume and history of whole brain radiation therapy.^(^ ^36^ ^–^ ^37^ ^)^ Also, a number of host‐related factors have been implicated in the mechanism of radiation necrosis including multiple brain surgical interventions and diabetes mellitus.^(^ ^41^ ^)^ The incidence of radiation necrosis has not been compared among GK and linac‐based stereotactic radiosurgical approaches. In this study, brain NTCP was negligible with both techniques, on the order of 10‐10 to 10‐8. However, radiation necrosis usually occurs within and around the tumor in the high radiation dose distribution. Whether the higher gEUDs delivered by GK or the slightly larger target volumes utilized by linac‐based stereotactic radiosurgery influence the incidence of radiation necrosis remains to be seen in studies of long‐term survivors.

In this era of cost‐containment, healthcare organizations need information about the cost‐benefit of a particular technology to make sound financial decisions. There are some studies on the cost‐effectiveness of GK versus linac‐based technologies.^(^ ^42^ ^)^ These indicate that unless there is significant patient volume to be treated with GK, the versatility of linac‐based technologies renders them a more attractive investment, especially for smaller radiotherapy centers.^(^ ^43^ ^–^ ^44^ ^)^ Cost‐benefit analysis has yet to be performed comparing GK with TOMO. However, with the ability of TOMO to treat both intra‐ and extracranial lesions, the omission of fixed framing, and the ability to deliver fractionated stereotactic radiation therapy, it may be prudent to assume a more favorable cost‐benefit ratio with such a system.

## V. CONCLUSIONS

In summary, stereotactic radiosurgery with GK results in superior tumor and normal brain dosimetry compared to TOMO. However, TOMO is useful for fractionated stereotactic radiotherapy, especially in cases with multiple brain lesions or those where concomitant brain radiation therapy is desirable. Clinical studies are needed to correlate the different dosimetric profiles of GK and TOMO with patient outcomes.
